# Spinal GABA transporter 1 contributes to evoked-pain related behavior but not resting pain after incision injury

**DOI:** 10.3389/fnmol.2023.1282151

**Published:** 2023-12-07

**Authors:** Bruno Pradier, Daniel Segelcke, Sylvia Reichl, P. K. Zahn, E. M. Pogatzki-Zahn

**Affiliations:** ^1^Department of Anesthesiology, Operative Intensive Care and Pain Medicine, University Hospital Muenster, Muenster, Germany; ^2^Department of Anesthesiology, Intensive Care and Pain Medicine, BG University Hospital Bergmannsheil, Ruhr-Universität Bochum, Bochum, Germany

**Keywords:** rat, GAT-1, NO711, plantar incision, intrathecal, multidimensional behavior

## Abstract

The inhibitory function of GABA at the spinal level and its central modulation in the brain are essential for pain perception. However, in post-surgical pain, the exact mechanism and modes of action of GABAergic transmission have been poorly studied. This work aimed to investigate GABA synthesis and uptake in the incisional pain model in a time-dependent manner. Here, we combined assays for mechanical and heat stimuli-induced withdrawal reflexes with video-based assessments and assays for non-evoked (NEP, guarding of affected hind paw) and movement-evoked (MEP, gait pattern) pain-related behaviors in a plantar incision model in male rats to phenotype the effects of the inhibition of the GABA transporter (GAT-1), using a specific antagonist (NO711). Further, we determined the expression profile of spinal dorsal horn GAT-1 and glutamate decarboxylase 65/67 (GAD65/67) by protein expression analyses at four time points post-incision. Four hours after incision, we detected an evoked pain phenotype (mechanical, heat and movement), which transiently ameliorated dose-dependently following spinal inhibition of GAT-1. However, the NEP-phenotype was not affected. Four hours after incision, GAT-1 expression was significantly increased, whereas GAD67 expression was significantly reduced. Our data suggest that GAT-1 plays a role in balancing spinal GABAergic signaling in the spinal dorsal horn shortly after incision, resulting in the evoked pain phenotype. Increased GAT-1 expression leads to increased GABA uptake from the synaptic cleft and reduces tonic GABAergic inhibition at the post-synapse. Inhibition of GAT-1 transiently reversed this imbalance and ameliorated the evoked pain phenotype.

## Introduction

The Gamma-aminobutyric acid (GABA)ergic system is an integral component of neuronal circuits also involved in processing sensory information. As a main inhibitory neurotransmitter, GABA is widely expressed in interneurons of the spinal cord and presents up to 40% of neurons in laminae I, II, and III (Polgár et al., 2003; Shimizu-Okabe et al., 2022). Inhibitory interneurons have various functions, and reduction of inhibitory GABAergic neurotransmission can cause disinhibition of excitatory transmission, which contributes to sensory hypersensitivity and spontaneous pain (Sandkühler, 2009; Comitato and Bardoni, 2021). Various mechanisms involving post-synaptic actions can lead to disinhibition (Kloc et al., 2019; Leonardon et al., 2022). Activating spinal GABA_A_ and GABA_B_ receptors inhibits mechanical and heat hypersensitivity (evoked pain-related), but does not alter non-evoked pain (NEP) in a model for post-surgical pain in male rats (Reichl et al., 2012). Furthermore, the altered spinal GABAergic tone is not associated with a change in expression of subunits of the spinal GABA_A_ (including α2 and α3) and GABA_B1/B2_- receptors (Reichl et al., 2012), which is in contrast to carrageenan-induced and neuropathic pain (Castro-Lopes et al., 1994; Ibuki et al., 1997).

GABA is synthesized via decarboxylation of L-glutamate through the enzyme glutamate decarboxylase (GAD with isoforms 65 and 67) in inhibitory interneurons (Soghomonian and Martin, 1998). Both isoforms are localized throughout the spinal cord, especially in the lamina I-III of the dorsal horn (Mackie et al., 2003) in GABAergic neurons. Both enzymes are involved in neuropathic pain-related behavior (Lorenzo et al., 2014; Wang et al., 2016) and may be responsible for chronic pain conditions (Vaysse et al., 2011). Furthermore, the functional loss of spinal GAD65 and/or GAD67 is associated with enhanced pain-related behavior via the reduction of tonic spinal GABAergic inhibition in the spinal cord (Vaysse et al., 2011; Zhang et al., 2011; Kami et al., 2016). After its release from synaptic vesicles, GABA is rapidly removed from the synaptic cleft through diffusion or GABA transporters (GAT). The most abundant isoform of GAT in the central nervous system is GAT-1, which is expressed in presynaptic neuronal structures and astrocytes (Yadav et al., 2015). Blockade of GAT-1 results in enhanced inhibitory GABAergic tone, leading to a longer circulation time and a higher concentration of GABA in the synaptic cleft and an increase in inhibitory post-synaptic potentials (IPSPs) (Bardoni et al., 2013). Genetic ablation of GAT-1 reduced pain responses after formalin injection (Xu et al., 2008), whereas GAT-1 overexpression has been associated with increased pain sensation (Ng and Ong, 2001). GABA uptake inhibitors such as tiagabine, (S)-SNAP-5114 or NO711 demonstrate potent analgesic effects in different animal pain models (Li et al., 2011; Kataoka et al., 2013; Zarêba et al., 2020; Gryzło et al., 2021; Oyama et al., 2022). However, the role of GAD65/67 and GAT-1 during post-surgical pain is still unclear. We hypothesized that inhibition of spinal GAT-1 would compensate the incision-induced imbalance in GABAergic signaling and thus ameliorate pain-related phenotype in a distinct way. Therefore, we comprehensively examined the impact of spinal GAT-1 inhibition on a number of different pain modalities post-incision and analyzed the expression levels of spinal GAT-1 and GAD65/67.

## Materials and methods

### General

**FIGURE 1:**
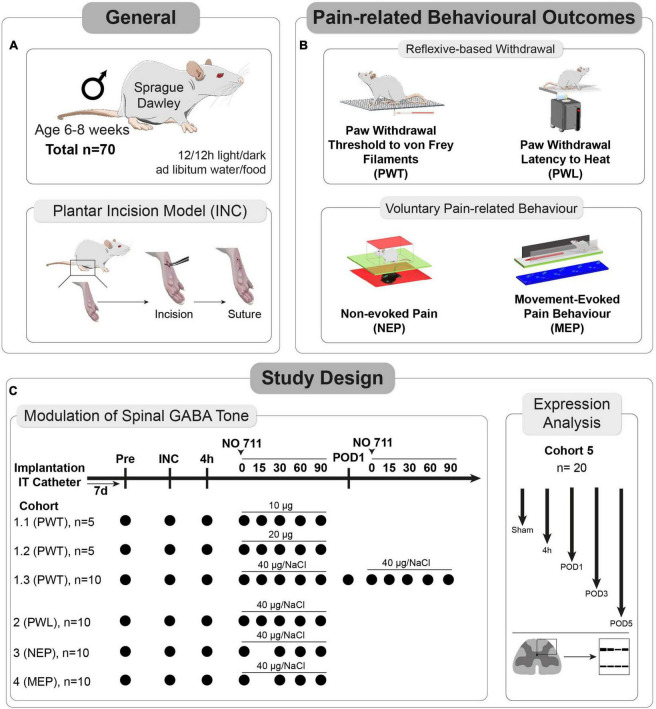
Study characteristics and design. **(A)** In total, 70 male Sprague Dawley^®^ (SD) rats, aged 6–8 weeks, and the plantar incision (INC) model to induce post-surgical pain were used for this study. **(B)** Pain-related behaviors were subdivided into two categories (1) reflexive withdrawal assays to mechanical (PWT) and heat (PWL) stimuli and (2) voluntary behavior, including movement-evoked pain behavior (MEP) and non-evoked pain behavior (NEP). **(C)** A total of 5 separate cohorts were used to determine the dose and timing for the *in vivo* application of a GAT-1 inhibitor (cohorts 1–4) and to investigate the time-dependent expression of GAT-1 and GAD65/67 in the dorsal ipsilateral spinal cord (cohort 5).

The experiments received approval from the Animal Ethics Committee of the State Agency for Nature, Environment, and Consumer Protection North Rhine-Westphalia (LANUV), Recklinghausen, Germany. Furthermore, they adhered to the recommendations outlined in the ARRIVE guidelines 2.0 (Du Percie Sert et al., 2020), and followed ethical guidelines for investigating experimental pain in conscious animals (Zimmermann, 1983). A total of 70 male Sprague Dawley^®^ (SD) rats, aged 6–8 weeks, were utilized in the study (see Figure 1A). Initially, the rats were housed in pairs in plastic open-top cages with a 12/12-h light/dark cycle, and they had unrestricted access to food and water.

After the catheter implantation procedure, the rats were individually housed. The allocation of rats to the experimental groups was done randomly, and a blinded analysis of video-based behavioral assessments was carried out. All individuals involved in conducting behavioral tests were unaware of the drug administration details. It should be noted that blinding for the withdrawal reflex-based behavioral assays and the pain model was not possible due to the testing conditions and visible indicators of the plantar incision, such as edema formation and sutures. At the conclusion of the experiments, all animals were humanely euthanized using an overdose of carbon dioxide.

### Pain models–general information

All procedures were performed on the right hind paw of the rats. Animals were initially anaesthetized with 5% isoflurane in 100% Oxygen; anaesthesia was maintained with 1.5–2.0% isoflurane delivered through a nose cone during the whole procedure. It’s important to note that no analgesics or antibiotics were administered during the procedure.

### Surgery induced post-surgical pain model–plantar incision model

Paw incision was based on the plantar incision model (Figure 1A) in rats (Brennan et al., 1996). To provide a brief overview, the entire right hind paw was first disinfected with 100% ethanol and Betadine^®^ (povidone-iodine). Subsequently, a longitudinal incision spanning the epidermis, dermis, fascia, and musculus digitorum brevis was made on the plantar aspect using a No. 11 scalpel (1 cm in length). The incision extended into the underlying muscle, which was temporarily retracted. Closure of the skin was achieved using two 5-0 Prolene mattress sutures, and the wound was covered with Betadine^®^ applied with a cotton swab. For the analysis of GABA-metabolism protein expression, sham-operated animals (subjected only to anesthesia) were included as a comparison group.

### Intrathecal catheter implantation

For the subarachnoidal application of the GAT-1 inhibitor NO-711, a lumbar intrathecal (IT) catheter was implanted (Pogatzki et al., 2000; Reichl et al., 2012, 2016). The procedure was carried out under general isoflurane anesthesia. Initially, the skin above the lumbar vertebrae was shaved and disinfected. A polyethylene tube, measuring 20 cm in length with an outer diameter of 0.61 mm and an inner diameter of 0.28 mm (Intramedic, Becton Dickinson, Franklin Lakes, NJ, USA), was carefully inserted into the spinal canal through a hypodermic needle positioned between lumbar vertebrae L5 and L6. It was advanced up to the level of the spinal cord between L4 and L5. After securing the catheter in place, it was tunneled in the cervical region, flushed with saline solution (0.9%), and sealed.

To confirm the accurate placement of the catheter, a small quantity (20 μl) of Xylocaine (2%) was infused into the rats after the behavioral experiments. Only those rats displaying a brief bilateral hind limb weakness following the Xylocaine injection were included in the analysis, ensuring the correct catheter localization.

## Multidimensional assessment of pain-related behaviors

### Reflex-based withdrawal behaviors for assessment of hypersensitivity at the ipsilateral hind paw

#### Punctate mechanical PWT

For the measurement of the withdrawal threshold (PWT) to punctual mechanical stimuli, rats were placed on an elevated mesh underneath a plastic box [dimensions 15 × 20 × 10 cm, HxWxD (Reichl et al., 2016)] and acclimatized for 30 min (Figure 1B). A withdrawal response was elicited using calibrated von Frey filaments with bending forces of 14, 20, 39, 59, 78, 98, 147, and 255 millinewtons (mN). These filaments were applied to the proximal part of the plantar aspect. The testing procedure began with a 14 mN force and progressed sequentially in increasing order until either a withdrawal response was observed or the maximum force of 255 mN (cut-off) was reached. The median value obtained from three separate series of tests determined the paw withdrawal threshold to mechanical stimuli. In instances where there was no withdrawal reaction to a particular filament, a force of 522 mN was considered as the response threshold.

#### PWL to heat

The withdrawal latency (PWL) to heat stimuli was determined using the Hargreaves apparatus (IITC, CA) as described recently (Figure 1B). Prior to conducting the PWL testing, the rats were individually placed on a heated glass surface set to a temperature of 30°C. This surface was enclosed by a transparent plastic box with dimensions of 15 × 20 × 10 cm (H × W × D) (Reichl et al., 2016), allowing the rats to acclimate for a period of 30 min. Subsequently, the beam of a heat lamp was directed toward the center of the incision area on the plantar surface. The intensity of the halogen lamp was adjusted to achieve withdrawal latencies of approximately 10–12 s under baseline conditions, with an intensity setting at 27% (Deuis et al., 2017). During the PWL assessment, a cut-off time of 20 s was established, and an inter-trial interval of 5–10 min was maintained. To determine the PWL, the mean values from three separate trials were calculated.

### Voluntary pain-related behaviors

#### MEP

Movement-evoked pain was assessed using the commercial CatWalk XT – System (Noldus, Netherlands) (Figure 1B) as described recently (Segelcke et al., 2022). Runs were included into analysis if animals presented a velocity range between 10 and 20 cm/s with a speed variance below 60%. These inclusion criteria enhance comparability across trials. The CatWalk XT system consists of an enclosed runway with a glass plate. In brief, the footprints on the runway are made visible by green light emitted from a LED strip inside the glass plate. The green light in the glass plate is internally reflected. Whenever the rat paws contact the glass, the green light is reflected downward to a high-speed camera (Illuminated Footprints™ technology). The total run is recorded by a high-speed camera (100 Hz) underneath the plate and evaluated with the CatWalk XT program. The rats are placed on the left end of the glass catwalk and should run into the goal box voluntarily three times, meeting previously defined parameters (speed variation < 60%, time under 5 s, speed between 20 and 25 cm/s). In this study, the following static (1,2) and dynamic (3,4) parameters (Kappos et al., 2017; Segelcke et al., 2021a,b) were used for gait analysis of MEP:

(1) Print area: area of the whole paw.
(2) Stand duration (s): ground contact duration for a single paw.
(3) Swing duration (s): duration of any swing cycle of a single paw.
(4) Swing speed (cm/s): time and distance at which a paw is not in contact with the glass plate.

#### NEP

Non-evoked pain (NEP) was assessed by comparing the weight-bearing (footprint area) of the injured (ipsilateral) paw with that of the uninjured (contralateral) paw while the rats were at rest, as previously outlined (Pogatzki-Zahn et al., 2021; Segelcke et al., 2021a,^2022^). To adapt the NEP measurement for rats, we followed a modified procedure originally designed for mice.

Briefly, rats were separately placed in transparent boxes (dimensions 15 × 20 × 10 cm, HxWxD) on a 1-cm-thick and green light illuminated glass plate. The boxes were covered by a slim LED panel (illuminated in red) to enhance contrast. Without prior habituation, images of the rats’ footprints were captured at 30-s intervals over a period of 10 min. The areas of illuminated footprints of both hind paws were blindly determined on ten different pictures for each rat using ImageJ (Schneider et al., 2012). The ratios of ipsilateral to contralateral illuminated hind paw areas were calculated for each time point and averaged for every animal. The selection of images for analysis adhered to predefined exclusion criteria, such as the presence of visible grooming, rearing, or instances where the hind paw was unclear due to movement. A reduction in the area ratios indicated guarding behavior of the injured limb while at rest.

### Protein expression analysis of GAT-1, GAD65, and GAD67 in the dorsal spinal cord

The expression levels of GAT-1, GAD65, and GAD67 in the dorso-ipsilateral spinal horn were assessed through Western blot analysis in a total of 20 rats at various time points (4 h, Postoperative day (POD) 1, 3, and 5) following the incision procedure, with sham-treated animals serving as the control group (as depicted in Figure 1C).

To carry out this analysis, tissue samples were first homogenized using RIPA buffer (Merck, R0278), and the resulting protein lysates were loaded onto an SDS-polyacrylamide gel. The proteins were then electrophoretically separated and transferred onto nitrocellulose membranes (Amersham-Bioscience, Freiburg, Germany). Subsequently, the membranes were blocked with 8% milk powder in TBS.

Antibodies specific to GAT-1 (AB1570W, anti-rabbit), GAD65 (ABA101, anti-rabbit), and GAD67 (MAB5406, anti-rabbit), all obtained from Chemicon (Merck-Millipore, US), were utilized at a dilution of 1:1000. As a loading control, β-actin (1:50,000, A5316, Sigma-Aldrich, Merck-Millipore, US) was employed. Signal intensities were detected using a digital system from BIORAD (Bio-Rad, US). The densitometric ratios of the GAT-1, GAD65, and GAD67 signals were then calculated relative to the loading control and expressed as a percentage in comparison to the control group (Sham).

### Spinal inhibition of GAT-1

To investigate the role of spinal GAT-1 in both evoked and non-evoked pain-related behaviors, we administered the GAT-1 inhibitor NO711 intrathecally (IT) in cohorts 1–4 (Figure 1C). NO711, obtained from Sigma-Aldrich (N142, CAS No 145645-62-1, Germany), was dissolved in 0.9% saline (NaCl, Vehicle). The IT injection volume amounted to 10 μl, followed by a 10 μl flush of preservative-free saline. The drug solution was freshly prepared on the day of each experiment.

**FIGURE 2:**
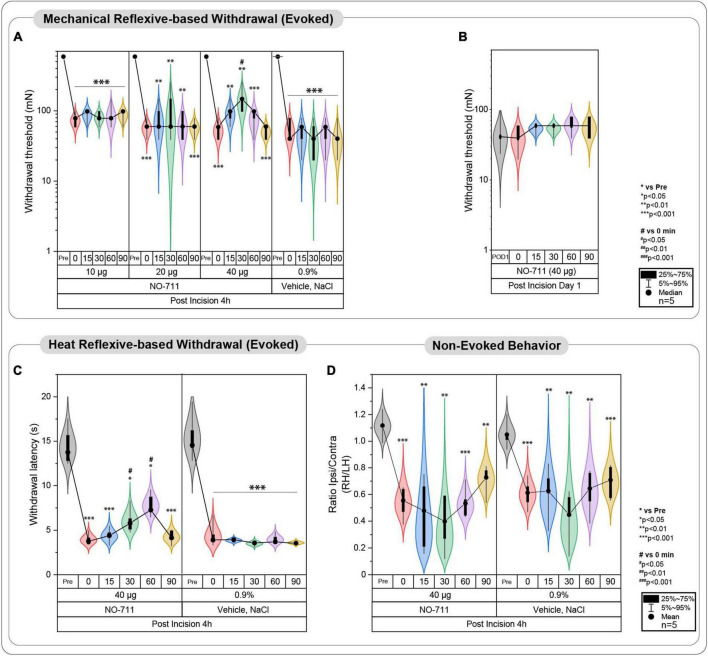
Evoked pain-related behavior is affected by spinal GAT-1 inhibition in the early phase post-incision injury. **(A)** Reflex-based withdrawal to mechanical stimuli (PWT) was determined in rats 4 h following incision or sham-procedure during inhibition of spinal GAT-1 with different doses of NO711 (10–40 μg) or vehicle (NaCl, 0.9%). Mechanical hypersensitivity was significantly reduced 30 min after spinal GAT-1 inhibition in a dose-dependent manner. **(B)** PWT was not altered in sham or incision rats and on postoperative day 1 (POD1) under inhibition of spinal GAT-1 with NO711 (40 μg). **(C)** Heat hypersensitivity (PWL) was also decreased following GAT-1 inhibition, 30 and 60 min after IT application of NO711 (40 μg). **(D)** Non-evoked pain behavior (NEP) was unaffected by the spinal inhibition of GAT-1. All experimental groups contain 5 mice (cohorts 1.1–1.3, 2, 3). The results are expressed as median ± 5–95% CI and Gamma curve type for panels **(A,B)**; mean ± 5–95% CI and Gamma curve type for panels **(C,D)**. *P*-values: *p < 0.05; **p < 0.01; ***p < 0.001 vs. Pre, ^#^*p* < 0.05 vs. 0 by Kruskal-Wallis and Dunnett’s multiple comparison tests.

In the initial phase, a group of 20 rats (cohorts 1.1–1.3) received intrathecal injections of NO711 at different doses (10, 20, or 40 μg) or a vehicle solution (0.9% saline) using a Hamilton syringe (with a volume of 50 μl) under tunnel handling conditions. Paw withdrawal thresholds (PWT) were assessed before the incision (Pre), 4 h after the plantar incision just before IT injection, and at 0, 15, 30, 60, 90 min following injection, as indicated in Figure 1C. In cohort 1.3, the impact of spinal GAT-1 inhibition (40 μg of NO711) was also evaluated 24 h after the incision. Subsequent experiments were conducted with the 40 μg dose of NO711 based on the results observed in cohorts 1.1–1.3, as this concentration notably reduced mechanical hypersensitivity following the incision (Figure 2A).

We exclusively employed animals that underwent incision in cohorts 1.1–1.3 for either NO711 or vehicle treatment. We deliberately excluded sham-operated animals from this experiment, as similar control experiments were previously conducted using the same intrathecal administration route (Li et al., 2011; Yadav et al., 2015). These prior investigations encompassed a range of NO711 concentrations from 5 to 100 μg and did not reveal any observable effects on the animals’ withdrawal thresholds. In alignment with the Animal Welfare Act and the 3R principles (Replacement, Reduction, Refinement), we made a conscious decision to minimize the number of animals in the current study and avoid duplicating the outcomes of previous reports. This rationale also guided our decision not to exceed a dosage of 40 μg for NO711 since we had already identified a significant drug effect.

Cohort 2 (PWT), cohort 3 (MEP), and cohort 4 (NEP) examined the effect of spinal GAT-1 inhibition (using NO711 at a dose of 40 μg) under the same experimental procedure described above.

### Statistic

The initial sample size calculation drew upon prior research employing the plantar incision model in male rats (Reichl et al., 2012, 2016). This calculation was carried out utilizing the G-Power 3.1.9.2 software.^1^ Subsequent to the power analysis, it was determined that a group size of 5 rats would provide an 80% statistical power to detect a change of 13%, assuming a significance level of 5%.

PWT raw data were analyzed by non-parametric analysis, such as Friedman test for within−group comparisons and the Kruskal–Wallis test for between−group comparisons. For PWL, NEP, and MEP behavior parameters, two-way ANOVA was used to analyze within- (to pre-value) and between-groups. Multivariate MEP behavioral data (cohort 3) were analyzed by principal component analysis (PCA) with prior standardizations (scaling data to have a mean of 0 and a SD of 1, PCs based on eigenvalues). PC selection was based on the largest eigenvalues. The first two principal components were plotted as bi-plots. Groups were added to the bi-plots for illustration but were not used during the PCA. The significance of group segregation was determined by multivariate analysis of PC loadings regarding the group with Tukey *post-hoc* tests. Multivariate ANOVA (MANOVA) was performed to provide regression analysis and analysis of variance for multiple dependent variables by one or more factor variables or covariates.

A significance level of *p* < 0.05 shows significant effects. Data were analyzed by Prism software, version 8 (GraphPad, USA), SPSS (IBM, USA) and OriginLab Pro 2023 (Originlab Corporation, US).

## Results

Unilateral incisions were used to elicit postoperative pain and were assessed by multidimensional behavioral tests in SD rats (Figure 1A). These included reflex-based withdrawal assays on the hind paw and approaches to assess voluntary pain-related responses (Figure 1B). Reflex-based behaviors were determined following paw withdrawal thresholds (PWT) to mechanical stimulation with von Frey filaments and paw withdrawal latencies (PWL) to heat stimuli, while voluntary pain-related behaviors were assessed during movement (movement-evoked pain, MEP) and while at rest (non-evoked pain, NEP). To investigate the role of the spinal GAT-1 on pain-related behaviors, we IT-applied different dosages of the GAT-1 antagonist, NO711, and tested animals of cohorts 1.1–1.3 at different time points post-incision (Figure 1C). In animals of cohort 1, we identified the best dosage (40 μg) of NO711 and time point (4 h) for further experiments with animals in cohorts 2–4. Finally, expression levels of GAT-1 and GAD65/67 were determined in the dorsal horn of the ipsilateral spinal cord to provide a mechanistic approach to the results from the behavioral experiments (cohort 5, Figure 1C).

### Spinal inhibition of GAT-1 ameliorates evoked- but not non-evoked pain 4 h post incision

Reflex-based withdrawal assays were used to determine thresholds to mechanical von Frey (PWT, Figures 2A, B) and heat stimuli (PWL, Figure 2C) at the ipsilateral hind paw. Incision injury significantly decreased mechanical thresholds at 4 h (Median ± Range; 78.45 ± 39.22 mN; *p* < 0.001 to pre; Figure 2A) and 24 h following surgery (39.23 ± 39.23; *p* < 0.001 to pre; Figure 2B). Blockade of spinal GAT-1 with lower doses (10 and 20 μg) 4 h following incision had no effect on PWT, while IT application of 40 μg of NO711 transiently reduced mechanical hypersensitivity 30 min following drug infusion (147.15 ± 158.92 mN; *p* < 0.05 to 0; Figure 2A). Interestingly, this effect was no longer present 24 h following incision injury (58.84 ± 19.61 mN; Figure 2B). Further, incision injury decreased PWL to heat stimuli 4 h following incision (Mean ± SD; 3.95 ± 0.63 s; *p* < 0.001 to pre; Figure 2C) and 30 (6.05 ± 1.36 s; *p* < 0.05 to pre) to 60 min (7.38 ± 1.24 s; *p* < 0.05 to pre). After spinal GAT-1 inhibition (NO711, 40 μg), PWL was significantly increased (*p* < 0.05 to pre) compared to the baseline (0 min). To test the impact of GAT-1 inhibition on non-evoked pain-related behavior at rest (NEP), we adapted our novel, video-based assessment established in mice (Pogatzki-Zahn et al., 2021; Segelcke et al., 2021a) to rats (Segelcke et al., 2022). In this assay, a decreased print area of the ipsilateral hind paw represents the NEP. NEP was significantly evident 4 h after incision (0.55 ± 0.12; *p* < 0.001 to pre) and was not modulated by spinal inhibition of GAT-1 compared to the baseline (0 min) (Figure 2D).

**FIGURE 3:**
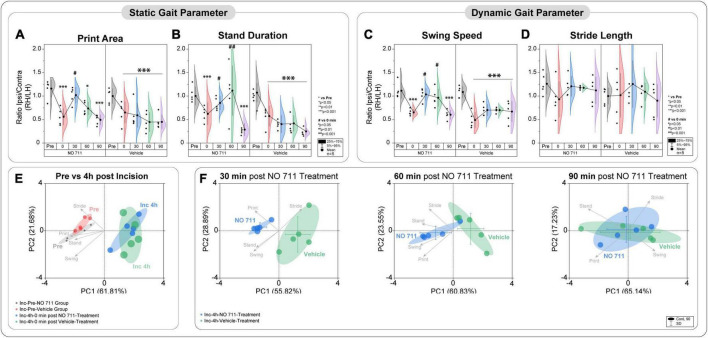
Spinal inhibition of GAT-1 ameliorates incision-induced movement-evoked pain. Gait analysis was performed using static **(A,B)** and dynamic **(C,D)** gait parameters. **(A–D)** Due to the unilateral incision, the print area, stand duration and swing speed, but not stride length, were reduced 4 h after the injury. Spinal inhibition of GAT-1 improved the incision-induced phenotype 30 and 60 min after NO711 application (40 μg). **(E,F)** Through the multivariate analysis of pain-related gait parameters, a distinct incision injury phenotype was found, and characterized by an antalgic gait pattern (reduction of stand time, print area, and swing time). This antalgic gait was time-dependently decreased by inhibition of spinal GAT-1, for 30 and 60 min after IT application of NO711 (40 μg), represented by significant group segregation compared to vehicle controls. The experimental group contained 5 mice (cohort 4). The results are expressed as mean ± 5–95% CI and Gamma curve type for panels **(A–D)**. *P*-values: *p < 0.05; ***p < 0.001. vs. Pre, ^#^*p* < 0.05; ^##^*p* < 0.01 vs. 0 by Kruskal-Wallis and Dunnett’s multiple comparison tests. **(E,F)** The PC components were selected to determine the eigenvalues. MANOVA was used for cluster analysis in PCA (Tables 1, 2).

In addition to NEP, movement-evoked pain (MEP) also represents a relevant symptom in post-surgical patients. We assessed static and dynamic ambulatory gait parameters during voluntary movement under standardized conditions through a video-based analysis. The static (print area, stand duration) and dynamic (swing speed) gait parameters are known to be altered in various unilateral pain models (Segelcke et al., 2021a,b). MEP was present 4 h after incision, as evidenced by significantly reduced print area (0.6 ± 0.22; *p* < 0.001 to pre), stand duration (0.61 ± 0.19; *p* < 0.001 to pre) and swing speed (0.57 ± 0.14; *p* < 0.001 to pre) (Figures 3A–C). As known from earlier studies, the incision did not change the stride length (0.98 ± 0.41) (Figure 3D; Segelcke et al., 2021a,b). Inhibition of spinal GAT-1 significantly reduced MEP (*p* < 0.05 to pre), 30 and 60 min after IT-application of GAT-1 inhibitor (NO711, 40 μg), compared to vehicle control (Figures 3A–C).

Using two-dimensional principal-component analyses (PCA), the antalgic gait phenotype was revealed by combining the static and dynamic gait parameters (Figures 3E, F). Principal-component (PC) scores and loadings revealed significant group segregation (*p* < 0.001 to pre) between two time points at baseline (Pre) and 4 h following incision injury for both groups (NO711, vehicle) prior to drug treatment. This effect was represented by directional loadings and distributed variance across both components (Figure 3E; Table 1). Both the static and dynamic gait parameters contribute equally to group segregation between Pre and post incision (4 h), with stand duration and print area having the most segregation in the x-direction. Inhibition of spinal GAT-1 reduced this antalgic gait phenotype, which was represented by higher loading of print area, stand time and swing time, 30 (*p* < 0.001 to Vehicle) and 60 min (*p* < 0.019 to Vehicle) following application of GAT-1 inhibitor (NO711, 40 μg) and by significant segregation from the vehicle-treated group (Figure 3E; Table 2). After 90 min, no significant group segregation was detected, as represented by unidirectional loading of all gait parameters (Figure 3E; Table 2).

**TABLE 1:** Cluster analysis of PCA results (Figure 3E) by multivariate analysis of variance (MANOVA).

Time	Model	Sig	Wilks/Lambda
Pre	NO711/Vehicle	0.809	0.973
4 h	NO711/Vehicle	0.653	0.948
Pre/4 h	NO711/Vehicle	<0.001***	0.128

***p < 0.001.

**TABLE 2:** Cluster analysis of PCA results (Figure 3F) by multivariate analysis of variance (MANOVA).

Time	Model	Sig	Wilks/Lambda
30 min	NO711/Vehicle	0.001***	0.101
60 min	NO711/Vehicle	0.019**	0.321
90 min	NO711/Vehicle	0.885	0.966

**p < 0.01; ***p < 0.001.

### Increase of GAT-1 and decrease of glutamate decarboxylase 67 (GAD67) expression in lumbal dorso-ipsilateral spinal cord 4 h after hind paw incision

**FIGURE 4:**
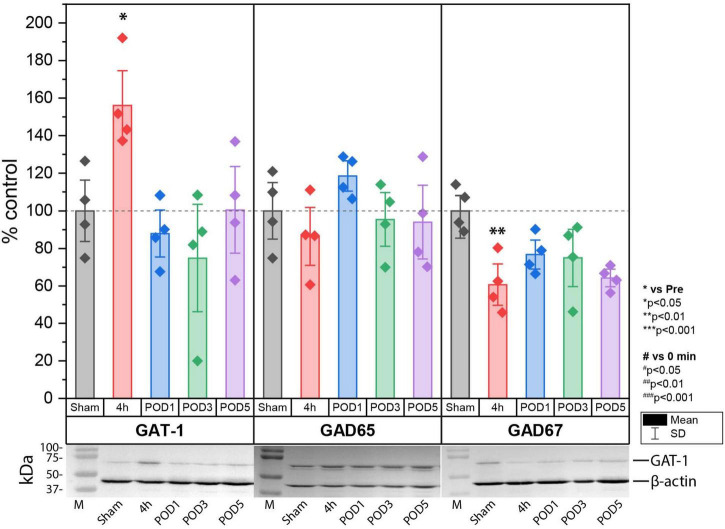
Protein expression of GAT-1 was significantly upregulated, while that of GAD67 was significantly downregulated in the spinal cord 4 h after incision compared to sham controls. Regulation at time points other than 4 h were not observed for GAT-1 and GAD67. No change in protein expression was found for GAD65. The expression is relative to β-actin and was normalized to the sham group and presented in single values in mean ± SD, *n* = 4 for each time point, *P < 0.05, **P < 0.01 vs. Sham by Tukey’s test. *M* = marker.

Next, we investigated the effect of incision injury on the expression of enzymes involved in the synthesis (GAD65 and 67) and synaptic transport (GAT-1) of GABA in the dorsal horn of the spinal cord (Figure 4). Protein expression was assessed in Sham control animals and at 4 h, POD1, POD3 and POD5 following incision injury in the ipsilateral dorsal horn. We found that 4 h after incision, the expression of GAT-1 (158.06% ± 24.71; *p* < 0.05 to sham) was increased while that of GAD67 (60.67% ± 14.79; *p* < 0.01 to sham) was decreased significantly compared to sham controls. At all other time points, no significant change in protein expression was detected. Moreover, the expression of GAD65 did not change after surgical incision.

## Discussion

In the present study, we identified a behavioral phenotype in male rats characterized by mechanical- and heat-hypersensitivity, an antalgic gait pattern, and non-evoked pain-related behavior in the early phase (4 h) following plantar incision injury in a model for post-surgical pain. At this time point, spinal inhibition of GAT-1 transiently ameliorated PWT, PWL, and MEP, but did not change NEP behavior. Furthermore, we observed an incision-induced transient increase of spinal expression of GAT-1 and a decrease of GAD67 ipsilateral to the incision.

The inhibitory GABAergic neurotransmission in the spinal cord is at the foundation of the gate control theory of pain (Melzack and Wall, 1965). Changes to this tonic inhibitory system, such as loss of spinal GABAergic neurons (Moore et al., 2002; Polgár et al., 2003), decrease of GABA synthesis/release (Wang et al., 2016), blockade of its receptors, or manipulation of GABA-transporters (Kataoka et al., 2013; Yadav et al., 2015; Zarêba et al., 2020; Gryzło et al., 2021), lead to disinhibition of spinal projection neurons and induce persistent neuropathic pain. Following incision injury, a role for GABAergic disinhibition has also been put forward. Accordingly, we previously described beneficial effects of GABA_A_ and of GABA_B_ receptor activation on evoked pain responses after incision in rats (Reichl et al., 2012). We could further show that modality-specific differences exist in other signaling pathways between non-evoked (pain at rest) and evoked pain after incision (Pogatzki-Zahn et al., 2017; Segelcke et al., 2019). For instance, we discovered that the glutamate transporter GLAST was involved in pain at rest rather than evoked pain responses (Reichl et al., 2016), while we found the opposite to be true for modulation of GABAergic signaling (Reichl et al., 2012). The activation of spinal GABA_A_ or GABA_B_ via IT-application of specific agonists ameliorated mechanical and heat hypersensitivity, but not NEP, represented by guarding behavior, at the day of the incision and on POD 1. This effect was not accompanied by a reduced receptor expression of GABA_A_ subunits α2 and α3 or the GABA_B_-receptor in the dorsal horn (Reichl et al., 2012). Therefore, other mechanisms might be responsible for changing the tonic GABAergic inhibition, as it has been suggested for other pain entities (Castro-Lopes et al., 1992, 1994). As indicated by others, apoptosis of spinal inhibitory interneurons (Moore et al., 2002; Scholz et al., 2005; Luo et al., 2016), but also trafficking of GABA receptors to or from the post-synaptic density might be involved, e.g., through gephyrin (Niwa et al., 2019). A further possible explanation for this effect is the regulation of the GABAergic tone via GABA transporter or GABA synthesis enzymes, which is directly related to *in vitro* studies that observed altered presynaptic GABA release probability upon prolonged excitatory stimulation (Fenselau et al., 2011; Leonardon et al., 2022). The clearance and homeostasis of extracellular GABA and, thus, maintenance of the necessary tonic GABAergic tone in the spinal cord for regulating the activity of projection neurons is modulated by GABA-transporters, especially GAT-1, which is expressed in presynaptic terminals and astrocytes (Ng and Ong, 2001; Kim et al., 2014; Yadav et al., 2015). In different pain models the expression of GAT-1 is disregulted in the spinal cord with inconsistent results. Most frequently, an increased GAT-1 expression or function was observed following inflammatory (formalin and carrageenan) insult or neuropathic pain in the spinal cord (Ng and Ong, 2001; Hu et al., 2003; Daemen et al., 2008) and Ncl. gracilis (Gosselin et al., 2010), while others showed reduced GAT-1 expression following chronic constriction nerve injury (Miletic et al., 2003). Such differences could arise from different temporal development of disease-specific signaling. For example, in previous neuropathic pain studies, many other examples for time-specific changes in signaling have been reported (Cobos et al., 2018; Segelcke et al., 2021a). In an earlier study, we identified a developmental variation in the function of cyclooxygenase 2 in postoperative pain (Segelcke et al., 2020), further reinforcing the link of function in development of pain disease. Here, for example, we described increased GAT-1 expression during the early phase 4 h following incision (initiation phase), but not at later time points (POD1, POD3 and POD5) during the maintenance phase, which would have been missed if only later time points had been investigated.

Our behavioral data demonstrate an amelioration of the evoked-pain (mechanical/heat hypersensitivity and antalgic gait pattern) only in the early time period (4 h) after incision and not at POD1. This analgesic effect is consistent with studies using acute heat stimuli (Hu et al., 2003; Kubo et al., 2009). Yet, it is surprising that NO711 does not exert analgesic effects at later time points. We previously demonstrated pronounced analgesic properties of muscimol at POD1 after incision injury in response to heat and mechanical stimulation (Reichl et al., 2012). Therefore, we consider the contribution of the present work as evidence on the pivotal role of GABAergic signaling in modulating only evoked pain responses. While both treatments increase the spinal GABAergic tone, they do so in different ways: with muscimol, a selective GABA_A_-receptor agonist is exogenously applied in addition to the endogenously available GABA, while NO711 increases levels of synaptically released intrinsic GABA, which then competes for binding at GABA_A_ and GABA_B_ receptors. We, therefore, speculate that muscimol specifically activates more GABA_A_ receptors at POD1 than does increasing the intrinsic concentration of GABA in the synaptic cleft by inhibiting GAT-1. Moreover, activation of both GABA receptor types, intracellularly could lead to activation of other signaling pathways than when only one GABA receptor type is activated. This may explain the differences in analgesic properties observed for both drugs at this time point.

Additionally, the absence of GAT-1’s effect at subsequent stages contrasts with findings from models of neuropathic, inflammatory, and bone cancer pain. In these models, inhibiting GAT-1 was found to regulate evoked pain-related behavior several days to weeks post initiation of experimental pain (Ng and Ong, 2001; Ford et al., 2015; Yadav et al., 2015; Luo et al., 2016; Zarêba et al., 2020; Oyama et al., 2022). This variation may signal specific differences related to the pain entity and could reveal previously unknown mechanisms of GABA metabolism that induce acute sensitivity to mechanical and heat stimuli shortly after an incision injury.

**FIGURE 5:**
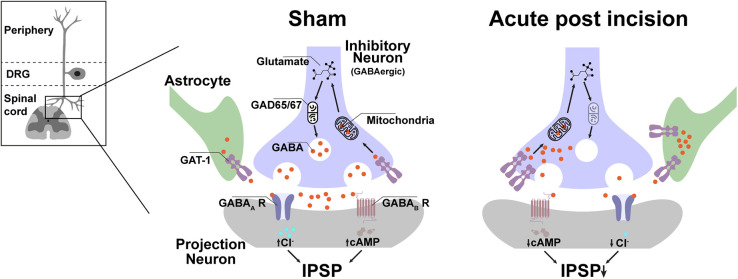
Possible spinal GABAergic metabolism disbalance plays a crucial role in evoked, but not non-evoked, pain-related behavior in the early phase after incision injury. In the acute period (4 h) after incision, GAT-1 expression in the lumbal dorso-ipsilateral spinal cord is increased, and GAD 67 is downregulated. The GAT-1 upregulation reduced the concentration and duration of GABA in the synaptic cleft and attenuated the GABAergic tonic effect to the projection neuron. This pathway seems to contribute to the formation of mechanical, heat hyperalgesia and movement-evoked pain but not to NEP. IPSP, inhibitory post-synaptic potential; GAD, Glutamate decarboxylase; cAMP, Cyclic adenosine monophosphate; Cl, chlorid; GABA, γ-Aminobutyric acid, GAT-1, GABA transporter 1; R, receptor.

Together, the rapid but transient increase of GAT-1 expression in the dorsal horn of the ipsilateral lumbal spinal cord is unique for incision injury and fits well together with the behavioral responses. The most likely scenario after incision is that the enhanced GAT-1 expression increases global GABA reuptake and contributes to the reduced GABAergic tonic inhibition of spinal projection neurons, which facilitates evoked pain after incision (Figure 5). The enhanced global reuptake of GABA in pre-synaptic terminals would further explain the reduction of GAD67 expression because new synthesis of GABA is not required and is therefore normalized at the same time as a GAT-1 expression.

In conclusion, the results of this study have identified GAT-1 as a promising target for perioperative pain management following incision injury. Specifically, a drug targeting GAT-1, which acts predominantly on evoked pain, would primarily benefit patients during post-operative activities, such as physiotherapy, or even basic tasks like sitting up or walking. In a clinical setting, a GAT-1 targeted drug might be most effectively used in combination with other analgesics that address spontaneous pain [e.g., glutamate transporter activation (Reichl et al., 2016)]. We hypothesize that the short-lasting increase of GAT-1 and decreased of GAD67 expression may represent an initial metabolic process in the formation of evoked pain after incision injury (hypersensitivity state). Further research is needed to reveal the role of GABA metabolism and the associated modulation of a spinal GABAergic system in postoperative pain conditions.

## Data availability statement

The original contributions presented in this study are included in this article/supplementary material, further inquiries can be directed to the corresponding author.

## Ethics statement

The animal study was approved by the Animal Ethics Committee of the State Agency for Nature, Environment and Consumer Protection North Rhine-Westphalia (LANUV), Recklinghausen, Germany. The study was conducted in accordance with the local legislation and institutional requirements.

## Author contributions

BP: Data curation, Investigation, Methodology, Visualization, Writing—original draft, Writing—review and editing. DS: Conceptualization, Data curation, Investigation, Methodology, Visualization, Writing—original draft, Writing—review and editing. SR: Conceptualization, Investigation, Writing—review and editing. PZ: Conceptualization, Methodology, Writing—review and editing. EP-Z: Conceptualization, Data curation, Supervision, Writing—original draft, Writing—review and editing.

## Footnotes

1
http://www.gpower.hhu.de/
